# Red flour beetle (*Tribolium castaneum*): From population genetics to functional genomics

**DOI:** 10.14202/vetworld.2018.1043-1046

**Published:** 2018-08-02

**Authors:** Harshit Kumar, Manjit Panigrahi, Supriya Chhotaray, V. Bhanuprakash, Rahul Shandilya, Arvind Sonwane, Bharat Bhushan

**Affiliations:** Division of Animal Genetics, ICAR - Indian Veterinary Research Institute, Bareilly - 243 122, Uttar Pradesh, India

**Keywords:** functional genomics, hox gene, insertional mutagenesis, RNAi, *Tribolium*

## Abstract

*Tribolium castaneum* is a small and low maintenance beetle that has emerged as a most suitable insect model for studying developmental biology and functional genetic analysis. Diverse population genetic studies have been conducted using *Tribolium* as the principal model to establish basic facts and principles of inbreeding experiments and response to the selection and other quantitative genetics fundamentals. The advanced molecular genetic studies presently focused on the use of *Tribolium* as a typical invertebrate model for higher diploid eukaryotes. After a whole genome sequencing of *Tribolium*, many areas of functional genomics were unraveled, which enabled the use of it in many technical approaches of genomics. The present text reviews the use of *Tribolium* in techniques such as RNAi, transgenic studies, immune priming, immunohistochemistry, *in situ* hybridization, gene sequencing for characterization of microRNAs, and gene editing using engineered endonuclease. In contrast to *Drosophila*, the *T. castaneum* holds a robust systemic RNAi response, which makes it an excellent model for comparative functional genetic studies.

## Introduction

Red flour beetles (*Tribolium castaneum*), in the order Coleoptera, is the very model of higher diploid animals. It is a flightless insect model came into the era of genetic studies back in the 1920s and served well in experiments of population genetics. *Tribolium* studies largely arose because of their ease in culture, relatively short generation interval, and status as globally distributed pests of stored grain and grain products. It carries ten pairs of chromosome in its genome, which makes it a simple yet suitable model for various genomic studies and helps revealing many unanswered theories of developmental biology and evolutionary studies. The lifespan of an adult insect is around 3 years, and the beetle is highly prolific reproducing after 1 month of age and producing on average 200–500 eggs in one biological life cycle. The temperature of about 27°C has been observed to be the most favorable for its development. The freshly hatched grub is small, worm-like, slender, cylindrical, and wiry in appearance. The body segments have a number of fine hairs, the terminal segment being in addition furnished with a pair of spine-like appendages. The full-grown grub is about 3/16′ long and is pale-yellowish in color. Sexing can be done at the pupal stage and not in larval forms for separating males and females. It infests stored food and cereals though, before the advent of storage of food grains, fungal infestation was suggested to be its habitat [1]. Extreme tolerance for hot, dry, and arid environments and contrasting expansion of genes asserting gustatory receptor explains *Tribolium’s* lifestyle of infesting animal food stores [2]. Thus, ease of handling, culturing, and maintenance of this organism in a laboratory made it a very popular experimental model in university research laboratories. Alexander Sokoloff documented exhaustive literature on this organism under three volumes and one monograph promoting the use of *Tribolium* in population genetics studies [3]. *T. castaneum* is the most extensively studied model for genetic studies right from the time of Chapman since then *Tribolium* remained in the limelight [4]. This species was also extensively studied for population differentiation, speciation, and host-parasite coevolution. Later on, it was used as a genetic tool for genetic mapping involving visible and molecular markers, balancing lethal mutations in true breeding stock using chromosomal rearrangements and transformation studies involving transposons. *Tribolium* is the only beetle whose complete genome sequence is available to date.

Modern-day genetics also envisage the use of *Tribolium* in transgenic studies and as a successful model for insertional mutagenesis [5]. In combination with tools available for transgenic manipulation, these techniques have established *Tribolium* as a powerful invertebrate model system for molecular genetics studies.

This review presents the findings and works carried out in *Tribolium* genetics since the 1920s, which extends from population studies to present-day advance molecular genetics and its future prospects.

## Tribolium and Population Genetics

Experiments in population genetics with *Tribolium* started with the work of Chapman in 1924, and hence, this beetle enjoyed a long history of being used as a model organism for population studies [4]. Beginning with basic culturing and sexing in the beetle, many researchers started breeding experiments with *Tribolium* and reported various findings related to population and metric genetics. Initially, population effects of inbreeding were studied. The flour beetles are also more appropriate laboratory models for research work in quantitative genetics for understanding gene action and its eventual usefulness in devising animal breeding programs, because of the occurrence of crossing over in both the sexes. Only limited reports on the influence of inbreeding on a few traits in this organism such as egg laying in Tribolium is available [6]. Effective population size along with the effects of inbreeding was reported on the mean and variance of the number of offspring in the next generation of flour beetle influenced by sex, genotype, and density [7]. Later, Wade *et al*. [7] reported the effect of inbreeding in response to the selection for the weight of pupa and heritable variance of fitness in *Tribolium*. Afterward, reports came for inbreeding effects on the redistribution of genetic variation of fecundity and viability in this beetle [6]. Recently, one group discovered inbreeding promotes female promiscuity [8]. The negative effect of inbreeding on phenotypic variance in the *T*. *castaneum* was reported by Pray and Goodnight [9].

## Tribolium in Classical Genetic Studies

As reported by Park [10], spontaneous mutations do not affect individual fitness, such as the eye-color mutation pearl, and hence, pearl eye mutation was used in differentiating *T. castaneum* and *T. confusum* more efficiently in population ecology experiments. During the year 1960, it was possible to develop genetic linkage maps for 7 autosomes and 1X chromosome. A minute region of chromosome 2 was later established as a focal point of deep and intense study succeeding the discovery of a single intact Hox gene complex by Beeman [11].

## Tribolium and Genomic Studies

It was back in 2008 when *Tribolium* genome sequence consortium gave a whole genome sequence of this beetle and later published in *nature* [2]. National Human Genome Research Institute (NHGRI), selected this beetle for whole genome sequencing because of its small size of the genome and simplicity of its organization. *Tribolium* genome size is bigger than *Drosophila* by 33% as well as in a number of genes, accounting almost 16,000 genes with 160 mb size of its genome. *Tribolium* surpasses *Drosophila* in every aspect of genomics studies, especially associated with embryonic development. When it comes to beetle, they account for around 25% of biodiversity share across the world and also share most of the genes with humans and livestock animals, which can be of principal interest to the researchers working with this beetle. After the uncovering of its genome sequence, many laboratories around the world are working on it and using this database in many technical methodologies associated with *Tribolium* genetic and genomic studies which are discussed further.

## Applications and Methodologies to Functional Genomics of Tribolium

### RNAi: Technical approach

RNA interference is a post-transcriptional mechanism of gene regulation, which is reported in all animals and plants including *Tribolium* and other insects. The implementation of RNAi in *Tribolium* and its use in studies inferring functional genomics laid novel avenues for insect genetic studies, like knockdown studies and also in insect pest management. RNAi can be done at various developmental stages of *Tribolium*. Females on treatment with dsRNA produce the suppression of gene expression in adults as well as in their progeny, a phenomenon called as “parental RNAi.” On changing, the dsRNA doses, a standardized series of genes, showing loss of functional phenotypic expression can be assembled. Red flour beetle shows unique and potent typical RNAi and often produces knockdown phenotypes, which cannot be differentiated from null mutants. Moreover, *Tribolium* enables fine-scale genetic analysis of genes functionally through its splice variant-specific RNAi [12]. In *Tribolium*, a large number of genes can be rapidly screened for specific knockdown phenotypes by injecting the corresponding double-stranded RNA (dsRNA), and in contrast to *Drosophila*, this approach is not restricted to early development. Since there is a lack of robust systemic RNAi response in *Drosophila* [13], *Tribolium* is insect model of choice specific for gene activity knockdown during different developmental stages.

#### Environmental RNAi

This refers to initiate RNAi by exposing dsRNA to the environment through soaking or feeding. Systemic movement of silencing signals may or may not follow environmental RNAi, hence a critical step in determining biological status of dsRNA. Components of the main systemic RNAi complex are easily identified in *Tribolium* since its genome is sequenced. *Tribolium* has been the excellent model for studying and understanding systemic silencing methods, whereas *L. decemlineata* (CPB) and *Diabrotica* (corn rootworm) are the emerging model systems for understanding the mechanism of the efficient environmental RNAi [14].

### In situ hybridization and immunohistochemistry

Most of the *in situ* and antibody staining methods and protocols used for *Tribolium* have been taken from studies conducted in *Drosophila*. Some of the few antibodies have been harvested specifically against *Tribolium* antigens also. The monoclonal antibodies mAbs4D9 and mAbs2B8 showed cross-reactivity with orthologous proteins from many varieties of insects, including *Tribolium* [15]. These two antibodies have been used extensively to define segmentation boundaries during early embryonic development.

### Transgenics

The methods and protocols for transgenesis in *Tribolium* genome are similar to that of *Drosophila*, which is also comparable in proficiency. Identification of transgenic beetles can be one by fluorescent protein expression mainly in the eye and brain tissues which are done by the artificial 3 × P3 promoter [16]. Other protocols are using EGFP or enhanced yellow fluorescent protein (EYFP) as the transformation marker. The latest technique involving this identification is Light sheet-based fluorescence microscopy [17].

### Insertional mutagenesis

In molecular biology, insertional mutagenesis is the creation of mutations of DNA by the addition of one or more base pairs. Such insertional mutations can occur naturally, mediated by viruses or transposons, or can be artificially created for research purposes in the laboratory. *Tribolium* insertional mutagenesis is similar to that of *Drosophila*. This is a two-component pattern system which uses base donor construct of piggyBac in the form of its mutagenic agent and a Minos-based helper construct to temporarily supply piggyBac transposase to mobilize the donor element [5]. The crossing of helper and donor strains leads to novel transposon insert which remobilizes the donor inserts [18]. Phenotypes of concern can be readily identified by molecular techniques from *Tribolium* genome, thus asserting readily identification of genes that are likely to be affected by transposons insertion. Thus, using these protocols and methods, a large-scale insertional mutagenesis project is established in the USA and Germany called the GEKU screen, and so far, they have generated more than 550 lethal and 600 enhancer trap lines (http://www.geku-base.uni-goettingen.de/).

### T. castaneum microRNAs (miRNAs): Identification by Solexa sequencing

miRNAs are short non-coding RNAs that regulate gene expression post-transcriptionally. They generally bind to the 3’-untranslated region of their target mRNAs and repress protein production by destabilizing the mRNA and translational silencing. As shown by whole genome locus analysis, *Tribolium*’s clusters of micro-RNAs are present in abundance. Erstwhile, few miRNAs of *Tribolium* were known, but this study revealed many new miRNAs. Knockdown of two genes, Dicer-1 and Argonaute-1, in late larval developmental stage impaired miRNA synthesis, and this further affected the hormonal pathways by upregulation of transcript levels in methoprene-tolerant and Krüppel homolog 1 (Kr-h1) leading to graded series of defects in the development and metamorphosis of *Tribolium*. Recently, Illumina or Solexa sequencing of eight critical developmental stages of *Tribolium* was conducted. In this study, 1154 unique miRNAs were reported, including 274 conserved miRNAs belonging to families of 68 miRNAs, 772 novel miRNAs, and 108 known candidate miRNAs [19].

### Long noncoding RNAs (lncRNAs) and immune priming in Tribolium

*T. castaneum* along with unique robust RNAi also possesses a memory-like mechanism termed as immune priming [20]. Immune priming is a mechanism by the innate immune response of invertebrate organisms for better protection against previously encountered pathogens. Many researches validated that this priming may occur over generation to generation, and then, it is referred to as transgenerational immune priming [18,21,22]. Recent studies investigating *T. castaneum* response to infection by *P. whitei* validated the relevance and importance of lncRNAs in the immune responses of *Tribolium* [15,23,24]. Further studies also evaluated lncRNA expression changes after *P. whitei* infection using long intervening noncoding RNA, i.e. lncRNAs located between two protein-coding genes that were annotated in *T. castaneum* [25].

### Tribolium and gene editing

Functional genomic studies in *Tribolium* gain a major boost with gene editing technologies since these are nothing but controllable mutagens. This enables the construction of null genes and any allele by stimulating DNA repair mechanisms using DNA sequence-specific endonuclease activity (Table-1) [26].

**Table-1:** Engineered endonuclease-based gene editing in *Tribolium* [26].

Insect	Author	date	Ref	Sys	Delivery	cells	genes	Mut	Results
Coleoptera, *T. castaneum*	Gilles	2015	63	C	Plasmid, mRNA, gRNA	S, G	EGFP	KO, KI	62–80% S KO 71–100% G KO 17% S KI 7%G KI

*T. castaneum=Triboliumcastaneum*

## Conclusion

*Tribolium* so far is the best eukaryote model for genetic studies, and this review highlights its use in dealing with many aspects of modern era genetics in the face of genomics. *Tribolium* certainly shows specialized traits of its own and possesses many ancestral developmental features (e.g., its manner of segmentation, the presence of external larval appendages, and non-involuted larval head morphology). These features, along with its sequenced genome, its susceptibility to RNAi, and the sophisticated genetic and transgenic methods that can be used on *Tribolium*, make this beetle an excellent choice for comparative studies. In the era of bioinformatics with various tools available, especially gene editing, *Tribolium* can easily establish itself as the best model for the same. This beetle has worthy potential in reverse genetics as well. In particular, its competence for systemic RNAi makes *Tribolium* better suited than *Drosophila* as a model organism for the study of certain basic biological problems, despite the highly advanced inventory of genetic tools available for the fruit fly.

## Authors’ Contributions

MP conceptualized and designed the manuscript. HK, SC, and RS prepared the manuscript draft and reviewed. RS contributed in literature collection. VB, AS, and BB edited and made critical comments on the manuscript. All authors read and approved the final version.
